# A novel genetic map of wheat: utility for mapping QTL for yield under different nitrogen treatments

**DOI:** 10.1186/1471-2156-15-57

**Published:** 2014-05-15

**Authors:** Fa Cui, Xiaoli Fan, Chunhua Zhao, Wei Zhang, Mei Chen, Jun Ji, Junming Li

**Affiliations:** 1 Center for Agricultural Resources Research, Institute of Genetics and Developmental Biology, Chinese Academy of Sciences, Shijiazhuang 050022, China; 2 State Key Laboratory of Plant Cell and Chromosome Engineering, Chinese Academy of Sciences, Beijing 100101, China; 3 University of Chinese Academy of Sciences, Beijing 10049, China

**Keywords:** Genetic map, Molecular marker, Quantitative trait loci, Wheat, Yield

## Abstract

**Background:**

Common wheat (*Triticum aestivum* L.) is one of the most important food crops worldwide. Wheat varieties that maintain yield (YD) under moderate or even intense nitrogen (N) deficiency can adapt to low input management systems. A detailed genetic map is necessary for both wheat molecular breeding and genomics research. In this study, an F_6:7_ recombinant inbred line population comprising 188 lines was used to construct a novel genetic map and subsequently to detect quantitative trait loci (QTL) for YD and response to N stress.

**Results:**

A genetic map consisting of 591 loci distributed across 21 wheat chromosomes was constructed. The map spanned 3930.7 cM, with one marker per 6.7 cM on average. Genomic simple sequence repeat (g-SSR), expressed sequence tag-derived microsatellite (e-SSR), diversity arrays technology (DArT), sequence-tagged sites (STS), sequence-related amplified polymorphism (SRAP), and inter-simple sequence repeat (ISSR) molecular markers were included in the map. The linear relationships between loci found in the present map and in previously compiled physical maps were presented, which were generally in accordance. Information on the genetic and physical positions and allele sizes (when possible) of 17 DArT, 50 e-SSR, 44 SRAP, five ISSR, and two morphological markers is reported here for the first time. Seven segregation distortion regions (SDR) were identified on chromosomes 1B, 3BL, 4AL, 6AS, 6AL, 6BL, and 7B. A total of 22 and 12 QTLs for YD and yield difference between the value (YDDV) under HN and the value under LN were identified, respectively. Of these, *QYd-4B-2* and *QYddv-4B*, two major stable QTL, shared support interval with alleles from KN9204 increasing YD in LN and decreasing YDDV. We probe into the use of these QTLs in wheat breeding programs. Moreover, factors affecting the SDR and total map length are discussed in depth.

**Conclusions:**

This novel map may facilitate the use of novel markers in wheat molecular breeding programs and genomics research. Moreover, QTLs for YD and YDDV provide useful markers for wheat molecular breeding programs designed to increase yield potential under N stress.

## Background

Common wheat (*Triticum aestivum* L.) has an allohexaploid genome (AABBDD, 2n = 6x = 42) with seven groups of homoeologous chromosomes, which complicates genetic and functional analyses in this species. The draft genome sequences of the wheat A-genome progenitor *Triticum urartu* and the D-genome progenitor *Aegilops tauschii* were recently released; these sequences can provide new insights into the A and D genomes and directly support map-based gene cloning [1,2]. However, accurate and detailed genetic maps are required for the molecular breeding of wheat and genomics research in this species.

A dense linkage map covering all 21 chromosomes is necessary for whole genome mapping in wheat; to produce such a map, various marker types should be combined. Microsatellites or simple sequence repeats (SSRs) are easy to use, exhibit a high degree of polymorphism, and frequently show co-dominant inheritance. Röder et al. [3] compiled the first g-SSR-based map of wheat, which included 279 loci. e-SSRs are derived from expressed genes, and their sequence information can be used to glean information on the function of the associated genes [4-6]. Gao et al. [7] reported the first wheat genetic map with 101 e-SSR loci. Inter-simple sequence repeat (ISSR) markers are reliable and share some of the advantages of microsatellites. Moreover, ISSRs have characteristics that are not species-specific [8,9]. However, to date, few ISSRs have been documented in wheat genetic maps [5].

Sequence-related amplified polymorphisms (SRAPs), which are based on open reading frames (ORFs) developed from genome sequence data of *Arabidopsis*, represent a novel PCR-based molecular marker technique [10]. SRAP targets functional genes and therefore can be efficiently used for purposes including gene tagging, marker-assisted selection (MAS), and genome-wide association studies [10,11]. Moreover, SRAPs have numerous other advantages such as multilocus and multi-allelic features, cost-effectiveness, and a lack of crop specificity. To date, few SRAP markers have been identified in wheat [5,11].

Diversity arrays technology (DArT) was developed as a hybridization-based alternative that captures the value of the parallel nature of the microarray platform [12]. This technique can generate hundreds of high-quality genomic dominant markers with high efficiency (http://www.diversityarrays.com/). Several wheat genetic maps that include DArT markers have been produced [12-19].

Wheat varieties that maintain yield under moderate or even intense nitrogen (N) deficiency can adapt to low input management systems. To breed such varieties, genetic variation for adaption traits to N deficiency is required. To date, limited quantitative trait loci (QTL) for both yield and its response to N deficiency in wheat under field conditions have been documented [20-22]. Detection of favorable alleles for yield that decrease difference between the value under high N (HN) and the value under lower N (LN) are of value in wheat breeding programs designed to increase N-deficiency tolerance.

In the present study, we develop a genetic map using a recombinant inbred line (RIL) population and compare this map with previously constructed physical maps. Genetic and physical positional information for more than 100 loci derived from SRAP, ISSR, e-SSR, and DArT markers are presented for the first time in this paper. Chromosomal regions harboring QTL for yield and yield sensitivity to N stress are specified, and we also inquire into their use in wheat molecular breeding programs. In addition, factors affecting the occurrence of segregation distortion regions (SDRs) and the total map length are discussed in depth.

## Methods

### Plant material and molecular markers

An F_6:7_ RIL population (denoted KJ) derived from a cross between Kenong9204 (KN9204) and Jing411 (J411) was used in this study. KN9204 was released in 2002 by the Center for Agricultural Resources Research, Institute of Genetics and Developmental Biology, Chinese Academy of Sciences, Hebei, China. As one of the representative cultivars in the North China Plain, it has higher yield potential and nitrogen use efficiency (NUE) than most other commercial cultivars [23,24]. The original RIL population contained 427 RILs. In this study, 188 randomly sampled lines from the 427 KJ-RILs were used for genetic linkage analysis and QTL detection.

The g-SSR, e-SSR, ISSR, STS, and SRAP molecular markers were used to genotype the parents and their derived lines. Information on g-SSR markers, including those with BARC, CFA, CFD, CFT, GWM, GDM, GPW, and WMC codes, and information on PCR-based STS markers with a MAG code was obtained from the GrainGenes website (http://wheat.pw.usda.gov). Relevant information on e-SSR markers with a CFE, KSUM, or CNL prefix is publicly available (http://wheat.pw.usda.gov/ITMI/E-SSR/). Information on e-SSR markers with the prefixes CWEM, EDM, CWM, CINAU, SWES, CAU, and BE/BF was published in reference articles by Peng and Lapitan [25], Mullan et al. [26], Gao et al. [7], Zhuang et al. [27], Li et al. [5,28], Yang et al. [29], and Lu et al. [30], respectively. ISSR markers were developed by the University of British Columbia Biotechnology Laboratory (UBCBL) [9]. Relevant information on SRAPs was obtained from an article written by Li and Quiros [10]. Polymorphic primer sequences for ISSRs and SRAPs are listed in (Additional file 1: Table S1). Information on DArT markers is publicly available (http://www.triticarte.com.au/). Information on the functional markers Ax2^*^, *Glu-b3h*, *PPO33*, and *STS01* was published in reference articles by Liu et al. [31]. The three functional markers *FM1*, *In10*, and *FM2* were developed by our research group and will be described in a forthcoming paper.

### Analysis of molecular/morphological markers and map construction

We used the touchdown PCR protocol described by Hao et al. [32] using a TaKaRa PCR thermal cycler (TaKaRa, Dalian, China). The amplification products were analyzed by polyacrylamide gel electrophoresis (PAGE), as described by Singh and Shepherd [33]. Seedling leaves were used to prepare DNA for DArT analysis following the recommended DNA extraction method (http://www.triticarte.com.au/content/DNA-preparation.html). All 427 RILs and their parents were assayed using the ‘Wheat *PstI* (*TaqI*) 2.3 D’ DArT array (the medium density array) (http://www.triticarte.com.au/). However, low DNA concentrations resulted in a limited number of polymorphic DArT markers.

The morphological markers *coleocolor* for the coleoptile color (green for KN9204, purple for J411) and *leaftype* for the flag leaf type (curling for KN9204, flat for J411) segregated as Mendelian factors (qualitative character inheritance) in the RIL population; these markers were phenotyped and assigned ‘A’ or ‘B’ scores for the linkage analysis.

The linkage groups were constructed using MAPMAKER 3.0 [34]. First, the ‘MAKE CHROMOSOME’ command was used to form 21 groups. Subsequently, the ‘ANCHOR’ command was used to assign SSR markers to their corresponding chromosomes using information from the publicly available genetic maps provided in GrainGenes 2.0 (http://wheat.pw.usda.gov/GG2/index.shtml). Anchor loci were chosen according to the following criteria: an absence of segregation distortion, minimal missing data, and optimal spacing along the chromosomes. The remaining markers were assigned to chromosomes using the ‘ANSIGN’ command at a log-of-odds (LOD) score of 2.5 and a distance of less than 45 cM. The ‘ORDER’ command determined the framework of each group. The ‘TRY’ command placed the remaining loci into the best fits (i.e., into the most likely intervals) of the corresponding groups at a LOD score of 2.0 and a distance of less than 50 cM. The ‘MAP’ command produced the linkage map. Lastly, the ‘RIPPLE’ command tested the robustness of each linkage map. The clusters that were identified as belonging to the same group were not linked if the distance between them was greater than 50 cM. The distances (in centiMorgans) were calculated using the Kosambi mapping function [35]. The map was drawn with MapChart 2.2 [36] (http://www.biometris.nl/uk/Software/MapChart/).

The observed segregation ratios were tested by chi-square analysis (1:1). A SDR was defined by at least three adjacent marker loci exhibiting a significant segregation distortion (P < 0.05). To validate the marker order of our genetic map, we compared the linear relationships between markers common to the new genetic map and to previously compiled physical maps [16,37-42]. For markers with discrepancies in chromosomal assignments and orders, we carefully checked the marker scores and re-estimated the positions of their corresponding loci.

### Field arrangement, trait evaluation, and QTL detection

The RILs and their parents were evaluated in Shijiazhuang in 2011–2012 (E1: 37°53′N, 114°41′E, altitude 54 m) and in 2012–2013 (E2), in Beijing in 2012–2013 (E3: 40°06′N, 116°24′E, altitude 41 m) and in Xinxiang in 2012–2013 (E4: 35°27′N, 113°48′E, altitude 95 m). Two nitrogen treatments were applied in each trial (LN and HN) for a total of eight environments (year × location × treatment) designated E1-LN, E1-HN, E2-LN, E2-HN, E3-LN, E3-HN, E4-LN and E4-HN. The soil nitrate-nitrogen (N) contents within the 0–20 cm layer in each environment are shown in (Additional file 1: Table S2). In each HN plot, 300 kg ha^−1^ of diamine phosphate and 150 kg ha^−1^ of urea were applied before sowing, and 150 kg ha^−1^ of urea were applied at the elongation stage every year. In the LN plots, no N fertilizer (N-deficient) was applied throughout the growing period. A randomized block design with two replications was used in each of the eight environments, and 40 seeds were hand-planted in each row of a two-row plot with 2-m long rows spaced 0.25 m apart. All of the recommended agronomic practices were followed in each of the trials except for the fertilization treatment described above.

For each plot, five representative plants in the center of each row were selected at physiological maturity to measure the yield per plant (YD). The difference between the value under HN and the value under LN in each trial was calculated as follows: *YDDV* = *YD*_*(HN)*_ –*YD*_*(LN)*_, where YDDV is the difference of the yield per plant for each line in each trial between the values under HN and that under LN, *YD*_*(HN)*_ and *YD(*_*LN)*_ represent YD under HN and LN, respectively. The broad-sense heritability (*h*_B_^2^) of the corresponding traits was calculated using the formula *h*_B_^2^ = *V*_*G*_/*V*_*P*_ in QGAStation 2.0 (http://ibi.zju.edu.cn/software/qga/v2.0/index_c.htm), where *V*_*G*_ and *V*_*P*_ are the genetic variance and phenotypic variance, respectively. The data from each environment were assembled individually according to the QTLData format of QGAStation 2.0. The first two columns represent the block (two replications) and genotype (the 188 lines), and the following columns represent YD and YDDV in each environment. The ‘environment effect’ ‘and block effect’ were attributed a value of ‘NO’ and ‘YES’ in ‘Ge Var’ analysis, respectively. To estimate the genetic by N treatment interaction variance (*V*_*G×T*_) for YD, the data from each trial (LN and HN) were assembled individually according to the QTLData format of QGAStation 2.0. The first three columns represent the treatment (LN and HN), block (two replications), and genotype (the 188 lines), and the following column was YD. Both the ‘environment effect’ ‘and block effect’ were attributed a value of ‘YES’ in ‘Ge Var’ analysis.

The inclusive composite interval mapping performed with IciMapping 3.3 (http://www.isbreeding.net/) was used to detect putative additive QTLs. For YD, the phenotypic values of the 188 RILs in E1-LN, E1-HN, E2-LN, E2-HN, E3-LN, E3-HN, E4-LN, and E4-HN were used for individual environment QTL mapping. Concerning YDDV, the phenotypic values of the 188 RILs in E1, E2, E3, and E4 were used for individual environment QTL mapping. The walking speed chosen for all QTLs was 1.0 cM and the *P*-value inclusion threshold was 0.001. The threshold LOD scores were calculated using 1,000 permutations with a type 1 error of 0.05.

## Results

### Molecular and morphological marker screening in the parental lines and in the 188 KJ-RILs

Polymorphisms between the KN9204 and J411 lines were found in 48.7%, 27.5%, 34.7%, 3.0%, and 3.7% of the primer pairs for the g-SSR, e-SSR, STS, ISSR, and SRAP markers, respectively. Using information from a survey of a high-density microsatellite consensus map [43], we selected 5–15 evenly distributed polymorphic g-SSR markers on each chromosome to produce the framework of the genetic map. For g-SSR markers, 174 primer pairs for 180 loci were polymorphic, including 63 WMC, 38 GWM, 35 BARC, 14 CFD, 11 GPW, seven GDM, four CFA, and one CFT code markers and 64, 38, 38, 14, 12, eight, four, and one loci, respectively. The primer pairs for *Xgdm88*, *Xwmc402*, and *Xgpw5215* each amplified two polymorphic loci, and the primer pair for *Xbarc1138* amplified four polymorphic loci. Forty-two polymorphic primer pairs for 50 loci of e-SSR markers were tested, including 14 CFE, seven KSUM, six CNL, six CAU, three SWES, two CINAU, one EDM, one CWEM, one BE, and one BF code markers and 14, 11, seven, seven, four, two, two, one, one, and one loci, respectively. The primer pairs for *Xcnl62*, *Xcau14*, *Xswes96*, and *Xedm149* each amplified two polymorphic loci, and the primer pair for *Xksum174* amplified five polymorphic loci. Fifteen STS primers with a MAG prefix for 17 loci were polymorphic, and only the primers for *Xmag2931* amplified two polymorphic loci. Polymorphism was also observed in 27 SRAP primer pairs for 46 loci and in three ISSR primers for six loci; each primer produced one to three polymorphic loci and an average of 1.73 and 2.0 loci. The seven primer pairs for the functional markers Ax2^*^, *FM1*, *Glu-b3h*, *In10*, *PPO33*, *STS01*, and *FM2* each produced their corresponding unique diagnostic fragments. The DArT analysis detected only 298 polymorphic DArT markers. The two morphological markers *coleocolor* and *leaftype* produced the qualitative inheritance of the relevant characteristic in the RIL population with a 1:1 segregation ratio, resulting in two morphological loci.

### The novel genetic linkage map

**Figure 1:**
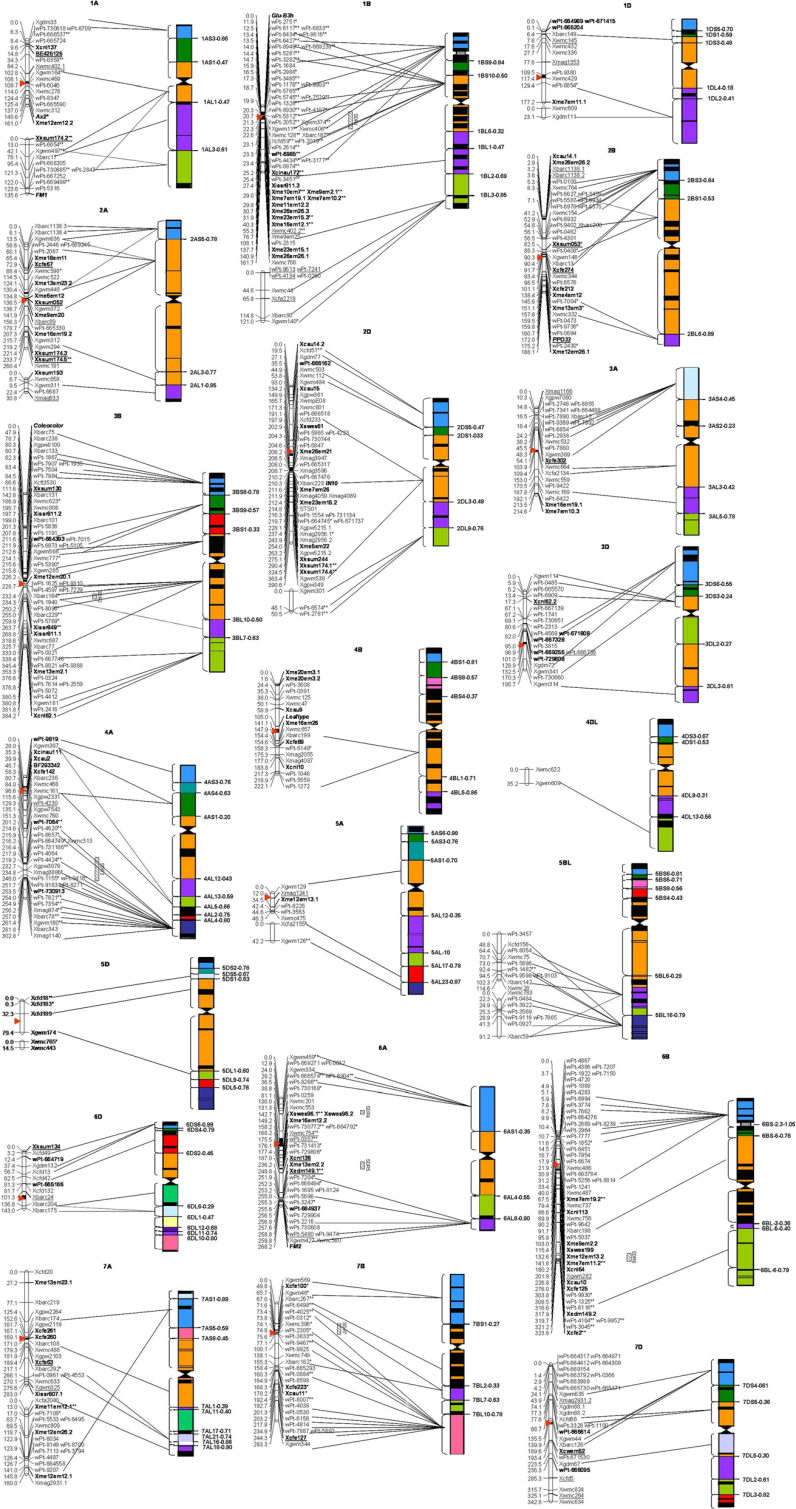
**Genetic map of wheat developed using an RIL population derived from the cross of the cultivars KN9204 and J411, and comparison of this novel map with previously developed physical maps of bread and durum wheat **[16,20-25]**.** The approximate positions of centromeres are indicated by *arrowheads*. Short arms are at the *top*. The positions of the marker loci are listed to the left of the corresponding chromosomes. The names of the marker loci are listed to the right of the corresponding chromosomes. *The lines* show the genetic/physical relationships between markers found on both the novel genetic map and the previously compiled physical maps. If the physical bin of a marker locus was inconsistent in more than one previous study, we indicated all of the possible physical positions of the locus. Markers that exhibited distorted segregation were marked by *(P < 0.05) or **(P < 0.01). Loci assigned to different chromosomes than those they were assigned to in previously compiled maps are underlined, and that are first reported in this map are marked by bold typeface.

Three PCR-derived polymorphic loci amplified by the primer pairs for *Xiss807*, *Xme7em10*, and *Xme12em20* and 11 loci detected by DArT analysis could not be assigned to chromosomes; these 14 polymorphic loci were therefore excluded. Moreover, 31 gaps that had a linkage distance greater than 40 cM but less than 50 cM were excluded from the count of the total map length. The genetic map based on the 188 KJ-RIL lines contained 591 loci on 21 wheat chromosomes and spanned 3930.7 cM, with an average density of one marker per 6.7 cM between the adjacent loci. Of the 591 loci, 287, 302, and two were DArT-based, PCR-based, and morphological markers, respectively (Additional file 1: Table S3; Figure 1). The primer sets that amplified two or more loci were mapped to homoeologous and non-homoeologous sites. The six functional markers Ax2^*^, *FM1*, *Glu-b3h*, *In10*, *STS01*, and *FM2* were accurately mapped to their corresponding chromosomes. *PPO33*, a functional marker for polyphenol oxidase (*PPO-A1*) on chromosome 2AL, was mapped to chromosome 2BL in this genetic map. The two morphological markers *coleocolor* and *leaftype* were mapped to the short arms of chromosomes 3B and 4B, respectively. Forty-one gaps greater than 40 cM in length were distributed across 17 chromosomes except 2B, 2D, 6B, and 6D (Figure 1). Nine gaps greater than 50 cM in length remained on chromosomes 1A, 1B, 1D, 2A, 2D, 5A, 5B, 5D, and 7A, whereas other chromosome arms were not covered (4DS, 5BS, and 6DL) (Figure 1).

Most markers were mapped to the B genome (44.8%) and A genome (33.5%), with an average of 37.9 and 28.3 markers per chromosome, respectively. The remaining markers (21.7%) were mapped to the D genome, with an average of 18.3 markers per chromosome. Although the map lengths for each genome were very similar, the chromosome sizes ranged from 35.2 cM (chromosome 4D) to 351.8 cM (chromosome 2D), with an average of 187.2 cM per chromosome. The number of markers on each chromosome ranged from two (chromosome 4D) to 61 (chromosome 1B), with a mean of 28.1 loci per chromosome. Chromosome 1B had the highest average marker density (one marker per 3.1 cM), and chromosome 4D had the lowest average marker density (average of 17.6 cM between adjacent loci) (Additional file 1: Table S3; Figure 1).

Forty-four (7.4%) of the 591 loci were assigned to chromosomes different from those to which the loci had been assigned in previous reports (Table 1) [27,31] (http://wheat.pw.usda.gov; http://wheat.pw.usda.gov/ITMI/E-SSR; http://www.triticarte.com.au). Interestingly, 21 (47.7%) of these loci were remapped to the corresponding homoeologous chromosomes of the previous studies. In addition, eight (18.2%) of these loci were reassigned to a chromosome that belonged to the same genomes (A, B, or D) as the chromosome to which they were assigned in the previous studies (Table 1).

**Table 1:** Markers that were assigned to different chromosomes in previous studies

***Marker***	***Previous***	***Present***	***Marker***	***Previous***	***Present***	***Marker***	***Previous***	***Present***
*BE425125*	7B	1AS	*Xksum174.5*	2D/4B	2AL	*wPt-0357*	6B	6AL
*Xwmc402.1*	7B	1AS	*Xmag633*	2D	2AL	*Xcnl138*	6B	6AL
*Xksum174.2*	2D/4B	1AL	*Xbarc1138.1*	2A/2D	2BS	*Xedm149.1*	5BL/6BL	6AL
*Xcinau172*	2D/3B/4D/1R	1BS	*Xbarc1138.2*	2A/2D	2BS	*wPt-5480*	6B	6AL
*Xwmc402.2*	7B	1BS	*Xksum053*	4B	2BS	*Xgwm282*	7A	6BL
*wPt-9613*	5B/7B	1BL	*Xcfe274*	4B	2BL	*Xksum134*	4A	6DS
*wPt-7241*	3D/7B/6R	1BL	*PPO33*	2AL	2BL	*Xbarc24*	6BL	6DS
*wPt-4134*	2B/3D/7B	1BL	*Xmag1166*	3B	3AS	*Xcfe63*	7B	7AL
*Xcfa2219*	1A	1BL	*Xcfe302*	3B	3AS	*Xgwm325*	5B/6B/6D	7AL
*Xwmc145*	6A	1DS	*Xksum130*	4A	3BS	*Xcfe127*	3B	7BL
*Xmag1353*	4A	1DS	*Xcnl62.2*	3B	3DS	*Xmag2931.2*	4A/7A	7DS
*Xcfe67*	2D	2AS	*wPt-666738*	3B	3DS	*Xcwem52*	7BL	7DL
*Xksum052*	7B	2AL	*wPt-4230*	6B/7B	4AL	*Xcfd5*	5B/5D/6D	7DL
*Xbarc89*	5BL	2AL	*Xmag1241*	2D	5AS	*Xwmc264*	3A/5D	7DL
*Xksum174.3*	2D/4B	2AL	*Xwmc754*	3B	6AL			

Relevant information on e-SSR markers with a CFE, CWEM, KSUM, or CNL is publicly available (http://wheat.pw.usda.gov/ITMI/E-SSR/). Relevant positional information on g-SSR markers, including those with a BARC, CFA, CFD, CFT, GWM, GDM, GPW, or WMC code, and on PCR-based STS markers with a MAG code, was obtained from the GrainGenes Web site (http://wheat.pw.usda.gov). Relevant information on DArT markers is publicly available (http://www.triticarte.com.au/). Information on the *Xcinau172* and *PPO33* loci was obtained from Zhuang et al. [27] and Liu et al. [31], respectively.

In total, 194 (32.9%) of the 589 molecular markers had previously been physically mapped to their corresponding chromosome bins [16,37-42]. The marker orders of loci from the present map were compared with those of previously published physical maps of bread or durum wheat. As shown in Figure 1, the orders were generally consistent. However, discrepancies in the marker orders were found on chromosomes 2BS, 3DS, 5BL, 7AL, 7BL, and 7DS.

### Novel molecular markers and their chromosomal location

Table 2 shows the chromosomal locations and genetic positions of the 17 novel DArT markers. These markers were assigned to chromosomes 1BS (one marker), 1DS (four markers), 2DS (one marker), 3BS (one marker), 3DS (four markers), 4AL (two markers), 6AL (one marker), 6DS (two markers), and 7D (two markers) by linkage analysis. We compared our genetic map with previously published physical maps and assigned 15 of these loci to 11 physical deletion bins (Figure 1, Table 2).

**Table 2:** Novel DArT markers and their chromosomal locations

***Marker***	***Chr.***	***Position***	***Physical bin***	***Marker***	***Chr.***	***position***	***Physical bin***
*wPt-6985*	1BS	23.3	1BS10–0.50	*wPt-729808*	3DS	101.0	3DS6-0.55–1.0
*wPt-664989*	1DS	0.0	1DS3-0.48–1.0	*wPt-7064*	4AL	201.2	4AL13-0.59–1.0
*wPt-671415*	1DS	0.0	1DS3-0.48–1.0	*wPt-730913*	4AL	253.5	4AL4-0.80–1.0
*wPt-665204*	1DS	0.1	1DS3-0.48–1.0	*wPt-664937*	6AL	255.6	6AL8-0.90–1.0
*wPt-666162*	2DS	35.5	2DS1-0.33–1.0	*wPt-664719*	6DS	12.4	6DS6-0.99–1.0
*wPt-664393*	3BS	211.6	3BS9-0.57–0.78	*wPt-665166*	6DS	81.3	–
*wPt-671808*	3DS	82.0	3DS6-0.55–1.0	*wPt-666614*	7DS	88.7	7DS4-0.61–1.0
*wPt-667328*	3DS	82.0	3DS6-0.55–1.0	*wPt-666095*	7DL	236.3	–
*wPt-669255*	3DS	96.9	3DS6-0.55–1.0				

‘–‘: No available data.

Fifty e-SSR-derived loci, 44 SRAP-derived loci, and five ISSR-derived loci were mapped for the first time on our map (Table 3, Figure 1). These markers were assigned to 18 chromosomes except 4D, 5B, and 5D. e-SSR primer pairs for KSUM174, CAU14, CNL62, and EDM149 each amplified multiple polymorphic loci that were mapped to homoeologous chromosomes. Two polymorphic loci amplified by the primer pairs for SWES96 were mapped to approximately the same position on chromosome 6AL. Sixteen (61.5%) of the 26 SRAP primer pairs amplified two or three polymorphic loci, and 45.7% of these loci were mapped to chromosomes that belong to the same genomes (A, B or D). Finally, 60.6% of the novel loci were assigned to their corresponding physical deletion bins based on the comparison of the present genetic map with previous physical maps (Table 3, Figure 1).

**Table 3:** Novel e-SSR, ISSR, and SRAP markers, their chromosomal location, and PCR products

***Marker***	***Chr.***	***Position (cM)***	***Alleles size (Kn9204/J411)***	***Physical bin***	***Marker***	***Chr.***	***Position (cM)***	***Alleles (Kn9204/J411)***	***Physical bin***	***Marker***	***Chr.***	***Position (cM)***	***Alleles (Kn9204/J411)***	***Physical bin***
*Xcnl137*	1AS	9.6	230 bp/240 bp	–	*Xme4em12*	2BL	138.4	185 bp/Null	–	*Xcnl10*	4BL	183.8	700 bp/710 bp	C–4BL5-0.71
*BE425125*	1AS	14.8	600 bp/Null	–	*Xme13em3*	2BL	151.1	410 bp/Null	2BL6-0.89–1.0	*Xme12em13.1*	5AS	11.8	Null/85 bp	–
*Xme12em12.2*	1AL	161.0	Null/210 bp	C–1AL1-0.47	*Xme12em26.1*	2BL	188.1	Null/85 bp	2BL6-0.89–1.0	*Xswes96.1*	6AL	142.7	400 bp/Null	–
*Xksum174.2*	1AL	0.0	Null/285 bp	C–1AL1-0.47	*Xcau14.2*	2DS	0.0	130 bp/Null	2DS5-0.47–1.0	*Xswes96.2*	6AL	142.7	Null/650 bp	–
*Xcinau172*	1BL	25.2	150 bp/180 bp	–	*Xcau15*	2DL	134.2	195 bp/200 bp	–	*Xme16em12.2*	6AL	149.2	397 bp/Null	–
*Xissr811.3*	1BL	27.4	Null/850 bp	1BL1-0.47–1.0	*Xswes61*	2DL	202.9	300 bp/Null	C–2DL3-0.49	*Xcnl138*	6AL	187.0	200 bp/203 bp	–
*Xme10em7*	1BL	29.0	200 bp/Null	1BL1-0.47–1.0	*Xme26em21*	2DL	206.2	Null/198 bp	C–2DL3-0.49	*Xedm149.1*	6AL	249.8	202 bp/Null	–
*Xme9em2.1*	1BL	29.0	Null/185 bp	1BL1-0.47–1.0	*Xme7em26*	2DL	211.6	150 bp/158 bp	C–2DL3-0.49	*Xme13em2.2*	6AL	236.2	160 bp/Null	–
*Xme7em19.1*	1BL	29.0	103 bp/Null	1BL1-0.47–1.0	*Xme23em15.2*	2DL	212.4	180 bp/Null	C–2DL3-0.49	*Xme7em19.2*	6BS	67.5	210 bp/Null	–
*Xme7em10.2*	1BL	29.0	110 bp/Null	1BL1-0.47–1.0	*Xme5em22*	2DL	254.0	103 bp/Null	C–2DL3-0.49	*Xcnl113*	6BS	86.6	250 bp/290 bp	–
*Xme11em12.2*	1BL	29.8	Null/386 bp	1BL1-0.47–1.0	*Xksum244*	2DL	275.1	Null/530 bp	C–2DL3-0.49	*Xme9em2.2*	6BL	103.0	Null/200 bp	–
*Xme26em26.3*	1BL	30.7	185 bp/Null	1BL1-0.47–1.0	*Xksum174.1*	2DL	290.4	500 bp/Null	C–2DL3-0.49	*Xswes199*	6BL	115.4	290 bp/300 bp	–
*Xme23em15.3*	1BL	31.9	400 bp/Null	1BL1-0.47–1.0	*Xksum174.4*	2DL	324.5	Null/300 bp	C–2DL3-0.49	*Xme12em13.2*	6BL	132.6	108 bp/Null	–
*Xme16em12.1*	1BL	40.3	Null/195 bp	1BL1-0.47–1.0	*Xcfe302*	3AS	54.1	285 bp/290 bp	–	*Xme7em11.2*	6BL	141.8	Null/180 bp	–
*Xme9em25*	1BL	76.7	210 bp/Null	1BL1-0.47–1.0	*Xme16em19.1*	3AL	213.5	98 bp/Null	3AL3-0.42–1.0	*Xcnl64*	6BL	180.2	550 bp/480 bp	–
*Xme23em15.1*	1BL	137.7	98 bp/Null	1BL3-0.85–1.0	*Xme7em10.3*	3AL	214.6	195 bp/Null	3AL3-0.42–1.0	*Xcau10*	6BL	226.8	160 bp/175 bp	–
*Xme26em26.1*	1BL	140.9	Null/85 bp	1BL3-0.85–1.0	*Xksum130*	3BS	111.6	150 bp/Null	3BS9-0.57–1.0	*Xcfe125*	6BL	276.0	428 bp/403 bp	–
*Xme7em11.1*	1DL	177.2	Null/160 bp	1DL2-0.41–1.0	*Xissr811.2*	3BS	197.7	380 bp/Null	3BS9-0.57–1.0	*Xedm149.2*	6BL	317.9	Null/200 bp	6BL5-0.40–1.0
*Xme18em11*	2AS	65.4	183 bp/Null	C–2AS5-0.78	*Xme12em20.1*	3BL	226.2	105 bp/Null	C–3BL10-0.50	*Xcfe2*	6BL	323.8	250 bp/254 bp	6BL5-0.40–1.0
*Xcfe67*	2AS	72.9	280 bp/300 bp	C–2AS5-0.78	*Xissr849*	3BL	263.7	720 bp/Null	C–3BL10-0.50	*Xksum134*	6DS	0.0	260 bp/269 bp	6DS6-0.99–1.0
*Xme13em23.2*	2AS	124.1	Null/420 bp	C–2AS5-0.78	*Xissr811.1*	3BL	268.8	280 bp/290 bp	C–3BL10-0.50	*Xme13em23.1*	7AS	27.2	200 bp/Null	–
*Xme6em12*	2AS	134.8	110 bp/Null	C–2AS5-0.78	*Xme13em2.1*	3BL	353.3	Null/150 bp	3BL7-0.63–1.0	*Xcfe261*	7AL	167.1	280 bp/305 bp	–
*Xksum052*	2AS	136.5	400 bp/450 bp	C–2AS5-0.78	*Xcnl62.1*	3BL	384.2	Null/480 bp	3BL7-0.63–1.0	*Xcfe260*	7AL	169.1	300 bp/Null	–
*Xme9em20*	2AS	141.9	492 bp/Null	–	*Xcnl62.2*	3DS	17.3	590 bp/585 bp	–	*Xcfe63*	7AL	189.4	380 bp/Null	–
*Xme16em19.2*	2AL	207.3	Null/280 bp	–	*Xcinau111*	4AS	28.0	160 bp/Null	4AS1-0.20–0.63	*Xissr807.1*	7AL	283	240 bp/Null	7AL21-0.74–1.0
*Xksum174.3*	2AL	221.4	292 bp/Null	2AL3-0.77–1.0	*Xcau2*	4AS	39.9	200 bp/206 bp	4AS1-0.20–0.63	*Xme11em12.1*	7AL	13.0	200 bp/Null	–
*Xksum174.5*	2AL	233.7	398 bp/Null	2AL3-0.77–1.0	*BF293342*	4AS	46.7	215 bp/Null	4AS1-0.20–0.63	*Xme12em26.2*	7AL	118.7	295 bp/Null	–
*Xksum193*	2AL	0.0	203 bp/201 bp	2AL3-0.77–1.0	*Xcfe142*	4AS	58.3	160 bp/155 bp	4AS1-0.20–0.63	*Xme12em12.1*	7AL	145.8	121 bp/Null	–
*Xcau14.1*	2BS	0.0	180 bp/175 bp	2BS3-0.84–1.0	*Xme20em3.1*	4BS	0.0	85 bp/Null	–	*Xcfe100*	7BS	49.8	470 bp/Null	7BS1-0.27–1.0
*Xme26em26.2*	2BS	2.9	Null/135 bp	2BS3-0.84–1.0	*Xme20em3.2*	4BS	1.6	Null/212 bp	–	*Xcfe223*	7BL	168.3	203 bp/200 bp	7BL10-0.78–1.0
*Xksum053*	2BS	82.5	Null/300 bp	C–2BS1-0.53	*Xcau9*	4BS	58.9	215 bp/205 bp	–	*Xcau11*	7BL	170.2	190 bp/185 bp	7BL10-0.78–1.0
*Xcfe274*	2BS	91.7	250 bp/Null	–	*Xme16em26*	4BL	141.1	Null/400 bp	–	*Xcfe127*	7BL	244.3	430 bp/Null	7BL10-0.78–1.0
*Xcfe212*	2BL	101.1	203 bp/209 bp	–	*Xcfe89*	4BL	154.6	450 bp/500 bp	C–4BL5-0.71	*Xcwem52*	7DL	189.6	251 bp/249 bp	–

‘–‘: No available data.

### Segregation distortion

On average, the 188 RILs inherited 49.5% of their alleles from the female parent (KN9204) and 50.05% from the male parent (J411) (data not shown). This result shows that the population was skewed in favor of J411 (*χ*^*2*^ = 8.8, P < 0.005). In total, 23.9% of the 591 loci significantly (P < 0.05) deviated from a 1:1 ratio, and 15.23% of the loci exhibited distorted segregations at the P < 0.01 level (Additional file 1: Table S4). Of these, 56 (60.3%) exhibited a segregation distortion in favor of J411 (P < 0.05). Additionally, the DArT markers exhibited a higher proportion of distortion (27.53%) than did the PCR-based markers (20.53%) (P < 0.05).

The marker loci with distorted segregation were not randomly distributed. The marker loci on genome B exhibited a higher proportion of distortion (31.7%) than did the loci on genomes A (23.2%) and D (8.6%) (Additional file 1: Table S4). Eighty-seven (61.7%) marker loci with distorted segregation were clustered in seven SDRs on chromosomes 1B, 3BL, 4AL, 6AS, 6AL, 6BL, and 7B (Additional file 1: Table S5; Figure 1).

### Phenotypic performance and QTL for yield and response to N stress

KN9204 showed similar YD to that of J411 in HN; in LN, the YD of KN9204 was higher than that of J411 (Table 4). In the 188 RILs, YD segregated continuously and generally followed a normal distribution in all environments, indicating that YD was a typical quantitative trait controlled by a few minor genes. The estimated broad-sense heritabilities of YD ranged from 33.32 to 82.95%, which showed higher value in HN than that in LN. G × T interaction was significant at the 0.01 level in all trials. In analysis of variance based on combined data in LN and HN, the genetic variance of YD accounted for 13.06–47.61% of the total variance. KN9204 showed lower YDDV than that of J411 in all environments, indicating that KN9204 has a higher yield potential in LN (Table 5). YDDV was revealed the feature of a typical quantitative character with estimated broad-sense heritabilities ranging from 71.24 to 81.86% (Table 5).

**Table 4:** Phenotypic performance for yield

***En.***^***a***^	***Trial***^***b***^	***Parents (g)***		***RIL***								
		**KN9204**	**J411**	**Mean (g)**	**Range**	**Skewness**	**Kurtosis**	**V**_**P1**_^**c**^	**V**_**G**_**/V**_**P1 **_**(%)**	**V**_**P2**_^**d**^	**V**_**G×T**_**/V**_**P2**_^**e **^**(%)**	**V**_**G**_**/V**_**P2 **_**(%)**
E1	LN	8.13**	7.24	6.57	7.68	0.27	1.18	2.11	33.32**	3.54	39.38**	20.31**
	HN	10.37	10.35	10.03	10.17	−0.01	−0.46	5.07	69.53**			
E2	LN	4.92**	3.62	5.58	7.57	0.40	−0.12	2.85	55.52**	7.53	58.22**	13.06**
	HN	14.30	14.28	13.28	18.74	0.31	0.37	12.22	74.93**			
E3	LN	9.87*	9.45	9.08	9.74	−0.31	0.72	2.77	50.75**	4.13	34.78**	37.19**
	HN	11.27	11.75	12.18	17.92	0.93	4.41	6.17	73.53**			
E4	LN	9.97	9.93	9.04	11.33	0.09	0.04	4.73	78.47**	5.73	33.69**	47.61**
	HN	11.65	11.85	10.93	14.11	0.04	−0.13	6.75	82.95**			

^a^En. = environments.

^b^LN and HN denote low nitrogen treatment and high nitrogen treatment, respectively.

^c^Phenotypic variance for yield in each of the individual trial of LN or HN.

^d^Phenotypic variance for yield in each of the combined trial of LN and HN.

^e^Genetic by N treatment interaction variance in each of the combined trial of LN and HN.

**Difference is significant when p < 0.01 level; *Difference is significant when p < 0.05 level.

**Table 5:** Phenotypic performance for yield difference between the value under HN and the value under LN

***En.***^***a***^	***Parents (g)***		***RIL***						
	**KN9204**	**J411**	**Mean (g)**	**Range (g)**	**V**_**P**_	**Std**	**Skewness**	**Kurtosis**	**V**_**G**_**/V**_**P **_**(%)**
E1	2.24	3.11	3.46	7.63	4.83	2.05	−0.21	−0.41	74.67**
E2	9.33	10.66	7.70	18.81	12.14	3.31	0.41	0.51	80.52**
E3	1.40	2.30	3.10	11.13	4.77	2.01	0.61	1.21	71.24**
E4	1.68	1.92	1.89	8.68	5.43	2.22	−0.20	0.50	81.86**

^a^En. = environments.

**Difference is significant when p < 0.01 level.

A total of 22 and 12 QTLs for YD and YDDV were identified and were distributed in 16 chromosomes except 5A, 4D, 5D, 6D, and 7D (Table 6). These QTL individually accounted for 3.93–26.64% of the phenotypic variance with LOD values ranging from 1.65 to 5.17. Of these, *QYd-4B.2* and *QYd-7B* showed significance in four of the eight environments, individually exhibiting 4.66–16.91% and 5.12–6.08% of the phenotypic variance, respectively. In addition, *QYd-3A* and *QYd-6B* were reproducibly detected in three of the eight environments; *QYd-1B.1* and *QYd-4B.1* were identified in two of the eight environments. The remaining QTL for YD showed significance in only one of the eight environments. Of these, *QYd-2D-1.1* was approximately 4.0 cM distal from *IN10*, one functional marker of *GS2* gene. For YDDV, only *QYddv-4B* was reproducibly detected in multiple environments, which individually accounting for 6.87–19.09% of the phenotypic variance with alleles from KN9204 decreasing YDDV (Table 6). It was noted that *QYd-1A.2-1* and *QYddv-1A.2*, *QYd-2A.1* and *QYddv-2A.1-1*, *QYd-4B-2* and *QYddv-4B*, *QYd-5B.2* and *QYddv-5B.2-1*, and *QYd-6A* and *QYddv-6A* were pairwisely co-located, respectively. For YD, there were 13 QTL (59.10%) with favorable alleles from KN9204 that increase YD. For YDDV, there were only two QTL (16.67%) with favorable alleles from KN9204 that decrease YDDV. Interestingly, alleles of *QYd-4B-2* from KN9204 increase YD in LN but decrease YD in HN, and therefore it decrease YDDV, which was verified by the negative additive effect of *QYddv-4B.*

**Table 6:** Putative QTL with significant additive effects for YD and YDDV

***QTL***^***a***^	***Position***^***a***^	***Flanking marker***^***a***^	***Trial***	***LOD***	***Add.***^***b***^	***PVE%***^***c***^
*QYd-1A.1*	15	*BE425125—wPt-6358*	E4-HN	2.82	−0.57	5.27
***QYd-1A.2-1***	**0**	***Xksum174.2—wPt-6654***	E1-HN	3.48	0.73	8.17
***QYddv-1A.2***	**0**	***Xksum174.2—wPt-6654***	E1	1.84	0.53	4.41
*QYd-1A.2-2*	121	*wPt-668205—wPt-667252*	E3-LN	1.85	−0.30	4.54
*QYd-1B.1*	23/28	*wPt-2751—wPt-2614*	E4-LN/E4-HN	4.33/1.99	−0.78/–0.42	8.54/4.03
*QYd-1D.1-1*	109	*Xmag1353—wPt-9380*	E4-LN	1.90	0.40	3.65
*QYd-1D.1-2*	177	*wPt-8854—Xme7em11.1*	E3-LN	2.03	0.33	4.91
***QYd-2A.1***	**124**	***Xwmc522—Xme13em23.2***	E3-HN	1.85	0.44	3.89
***QYddv-2A.1-1***	**130**	***Xme13em23.2—Xgwm448***	E2	1.95	0.69	4.31
*QYddv-2A.1-2*	164	*Xbarc98—wPt-665330*	E3	1.81	0.53	6.82
*QYddv-2B*	6	*wPt-0100—Xwmc764*	E3	2.10	−0.45	5.10
*QYd-2B*	86	*Xksum053—wPt-0408*	E1-HN	2.50	0.56	8.17
*QYddv-2D.1*	77	*Xwmc112—Xgwm484*	E4	1.85	0.77	**11.93**
*QYd-2D.1-1*	206	*Xmag3956—IN10*	E2-LN	2.21	0.34	5.27
*QYd-2D.1-2*	332	*Xksum174.4* ***—*** *Xgwm539*	E2-LN	2.01	0.50	**10.81**
*QYd-3A*	103/100/120	*Xcfe302—Xwmc559*	E2-HN/E4-LN/E4-HN	1.68/2.63/3.16	0.56/0.85/1.02	8.32**/17.03/16.63**
*QYd-3B*	117	*Xksum130—Xbarc131*	E4-HN	2.43	−0.64	6.69
*QYd-3D*	5	*Xgwm114—wPt-0485*	E3-HN	3.24	−0.61	7.30
*QYddv-3D-1*	68	*wPt-1741—wPt-730651*	E4	2.11	0.51	5.33
*QYddv-3D-2*	100	*wPt-666738—wPt-729808*	E4	2.12	0.51	5.34
*QYd-4A-1*	47	*BF293342—Xcfe142*	E2-LN	2.12	0.42	4.06
*QYd-4A-2*	103	*Xwmc161—Xgpw2331*	E1-LN	2.42	−0.34	8.66
*QYd-4B-1*	50/54	*Xwmc125—Xcau9*	E4-LN/E4/HN	4.91/3.57	0.65/0.70	**10.01**/7.90
***QYd-4B-2***	**202/184/188/181**	***Xwmc657—wPt-1046***	E2-LN/E2-HN/E3-HN/E4-LN	2.61/2.94/2.23/2.15	0.62/–0.87/–0.60/0.44	**16.91**/7.06/7.03/4.66
***QYddv-4B***	**190/174**	***Xwmc657—wPt-1046***	E2/E3	5.17/5.05	−1.45/–0.45	**19.09**/6.87
***QYd-5B.2***	**0**	***Xwmc783—wPt-0408***	E1-HN	1.96	0.43	4.29
***QYddv-5B.2-1***	**0**	***Xwmc783—wPt-0408***	E1	1.85	0.43	4.44
*QYddv-5B.2-2*	42	*wPt-0927—Xbarc59*	E2	2.31	0.75	5.13
***QYd-6A***	**259**	***wPt-9474—Xwmc580***	E2-LN	1.89	−0.32	4.54
***QYddv-6A***	**236**	***Xcnl138—Xme13em2.2***	E3	1.89	0.43	4.60
*QYd-6B*	22/22/22	*Xwmc486—wPt-663764*	E2-LN/E4-LN/E4-HN	1.70/2.54/2.25	0.38/0.46/0.49	4.25/5.04/3.93
*QYddv-7A.2*	39	*wPt-7108—wPt-6495*	E1	1.89	1.06	**26.64**
*QYd-7A.2*	150	*Xme12em12.1—Xmag2931.1*	E4-LN	3.15	−0.60	8.52
*QYd-7B*	75/80/76/73	*Xcfe100—wPt-9467*	E2-HN/E3-LN/E3-HN/E4-LN	1.52/2.05/2.77/1.65	0.30/0.36/0.58/0.31	5.01/6.08/6.30/5.12

^a^YD: Yield per plant; YDDV: yield difference between the value under HN and the value under LN. Co-located QTL for YD and YDDV was marked by bold typeface.

^b^PVE: Phenotypic variance explained by the corresponding putative additive QTL; PVE% that was > 10% was marked by bold typeface.

^c^Additive effect of the corresponding putative additive QTL; positive values indicate KN9204 alleles that increase the value of the corresponding trait, and, conversely, negative values indicate KN9204 alleles that decrease it.

## Discussion

### The total map length and marker distribution across the wheat genome

Numerous molecular genetic maps covering the entire hexaploid genome of wheat have been compiled (Additional file 1: Table S6). These maps have described the hexaploid wheat genome as encompassing genetic distances ranging from 2260 cM to 5332 cM. Sourdille et al. [38] suggested that the hexaploid wheat genome encompasses approximately 4000 cM in the case of an intraspecific population. Certain studies have confirmed this finding, whereas other studies have produced maps covering <3500 cM (Additional file 1: Table S6). Interestingly, Gao et al. [7] developed an integrative map with a total length of 4641 cM, and Ganal and Röder [43] produced an integrative map encompassing 5332 cM of the wheat genome. Nachit et al. [44] produced a durum wheat (AABB, 2n = 4X = 28) map spanning 3598 cM using an intraspecific RIL mapping population. These reports imply that it is possible to produce a molecular genetic map spanning >5000 cM for allohexaploid bread wheat using a large intraspecific population and various types of molecular markers.

In the present study, we produced a novel molecular genetic map for wheat based on various types of molecular and morphological markers. This map encompassed 5257 cM of the wheat genome, including 31 gaps with a linkage distance greater than 40 cM but less than 50 cM. This map length corresponded to that of an integrative map reported by Ganal and Röder [43]. To the best of our knowledge, this is the longest molecular genetic map of common wheat based on an individual intraspecific mapping population. The moderate mapping population size (188 lines) and the variety of molecular markers that were mapped might explain the completeness and length of this genetic map. Including the 31 large gaps with a distance of more than 40 cM would have resulted in an overestimation of the total map length, as the LOD values for pairwise loci with a distance of >40 cM were relatively low (approximately 2.5). Therefore, when excluding the 31 large gaps from the count of the total map length, we produced a map with a total length of 3931 cM, which is comparable to the length of the map produced by Sourdille et al. [38].

A lower number of polymorphisms in the D genome than in the A and B genomes has been documented and is consistent with the hypothesis of a monophyletic introduction of the D genome in bread wheat [45]. Almost all of the previously compiled genetic maps of allohexaploid bread wheat listed in the additional file (Additional file 1: Table S6) mapped fewer marker loci in the D genome than in the A and B genomes, especially on chromosome 4D. In the present map, the D genome contains only 21.7% of all loci, and chromosome 4D contains only two g-SSR markers, which is consistent with the findings of previous reports. However, the achieved map coverage was almost identical for the three genomes, which is also consistent with previous reports.

### Comparison of the present genetic map with previous physical maps

To date, many molecular markers, including RFLP, g-SSR, e-SSR, STS, and DArT markers have been physically mapped [16,37-42]. One hundred ninety-four (32.9%) of the 589 molecular markers mapped in the present study had previously been physically mapped to their corresponding chromosome bins; this information allowed us to evaluate the colinearity of the markers and thus directly support fine mapping of major QTLs and map-based gene cloning.

Although the chromosome assignments produced in this study are largely in agreement with previously published mapping information, discrepancies remained for 44 (7.4%) of the 591 loci (Table 1). Of these, 21 (47.7%) markers were mapped at positions homoeologous to the positions reported in previous maps. In addition, eight (18.2%) loci were mapped to chromosomes that belonged to the same genomes (genomes A, B, or D) as the chromosome to which these loci were mapped in previous studies. The relative order of markers within linkage groups in the present map was largely consistent with the order of markers found in previously published physical maps. However, discrepancies remained for chromosomes 2BS, 3DS, 5BL, 7AL, 7BL, and 7DS (Figure 1). Inaccurate genotyping for these markers were excluded by carefully checking the marker scores. However, these discrepancies remained. These markers might be multilocus markers, and the novel polymorphic sequence on other chromosomes or the same chromosome was detected and mapped in this population. In addition, chromosomal rearrangement such as interchromosomal/intrachromosomal translocation or paracentric inversion might explain these discrepancies. Further studies are necessary to confirm this hypothesis.

A vast majority of recombination events occur on the most distal portions of the chromosomal arms in wheat; recombination events around the centromere tend to be suppressed [37,46]. Previous studies have supported the idea that large genetic distances in the centromeric region on genetic maps correspond to small genetic distances on physical maps [16,37-42]. A comparison of the present genetic map with previously published physical maps confirmed this conclusion. For example, four markers spanning >170 cM in the distal portion of chromosomal arm 1BL accounted for only 15% of the length of the 1BL arm; nine markers spanning approximately 50% of the distal portion of the 2BS arm in the genetic map accounted for only 16% of the 2BS arm in the physical map; and fourteen markers spanning approximately 37% of the distal portion of the 4AL arm in the genetic map accounted for 20% of the 4AL arm in the physical map. Finally, twelve markers spanning approximately 90% of the distal portion of the 7BL arm in the genetic map accounted for only 22% of the 7BL arm in the physical map (Figure 1).

### Novel markers and multilocus markers

e-SSRs are more transferable between species than microsatellites derived from genomic libraries [6,47]. SRAPs and ISSRs have characteristics that are not species specific. Moreover, e-SSRs and SRAPs are generally directly related to functional genes [9,11]. To date, few SRAP and e-SSR markers have been integrated into wheat SSR maps [4,5,7,48]. In the present study, we provided detailed information on the chromosomal locations of 17 DArT, 50 e-SSR, 44 SRAP, and five ISSR markers (Tables 2, 3; Figure 1). We also presented the positions and orders of these markers relative to those of the g-SSRs, in addition to the size of the allele in the two mapping parents and the estimated corresponding physical bins when possible (Table 3). This information facilitates the use of these markers in wheat molecular breeding programs and genomics research.

Aneuploidy and linkage analyses have previously shown that the three genes controlling the red coleoptile color (*Rc1*, *Rc2* and *Rc3*) are located on chromosomes 7A, 7B, and 7D, respectively [49-52]. The morphological markers for the coleoptile color (the *coleocolor* locus) and flag leaf type (the *leaftype* locus) segregated as Mendelian factors in the RIL population and mapped to 3BS and 4BS, respectively (Figure 1). Therefore, the *coleocolor* locus may harbor a novel gene controlling the coleoptile color. To the best of our knowledge, no other reports have mapped the genes that control the flag leaf type to the 4BS chromosome arm of wheat.

Although SSR and STS markers are believed to be locus-specific, several primer pairs amplified more than one fragment in this study [3,53]. It is noted that multiple loci identified by certain e-SSR, g-SSR, and STS markers in allohexaploid wheat were apt to be mapped to homoeologous positions. This finding is consistent with the polyploidy, synteny and homoeology of the three genomes (A, B, and D) [54-56]. In total, 45.7% of the 44 loci amplified by 16 SRAP primer pairs were pairwisely mapped to chromosomes that belong to the same genome (A, B or D) rather than to homoeologous positions. The remaining loci amplified by specific SRAP primer pairs were scattered among the wheat genomes (Table 3, Figure 1). As an ORF-based marker system that targets functional genes, SRAPs have multilocus, multi-allelic, and non-specific characteristics among genomes and species [11]. These features might explain the abovementioned findings.

### Segregation distortion

Segregation distortion is a common phenomenon that can be influenced by chromosomal rearrangements, alleles inducing gametic or zygotic selection, parental reproductive differences, the presence of lethal genes, and environment factors [44,49,57-60]. A biological segregation distortion locus can cause the locus of interest and the flanking regions to deviate from the expected Mendelian segregation ratio, thus forming an SDR [57,61,62].

In the present study, seven SDRs on chromosomes 1B, 3BL, 4AL, 6AS, 6AL, 6BL, and 7B were identified; these SDRs might have been produced by biological factors (Additional file 1: Table S5). Previous studies have also reported the presence of SDRs in these chromosomal regions [15,44,48,49,56,63]. It is noteworthy that 42 (29.8%) loci with distorted segregation were clustered on the SDR1 of chromosome 1B, which may be related to 1RS in parental line KN9204. Approximately 38% and 62% of the 188 RILs contain 1RS and 1BS, respectively, which is consistent with our previous three RIL populations (WL, WY, and WJ) [19]. This finding suggests that the normal 1B chromosomes are more capable of being preferentially transmitted through the male and female gametes than are the unbalanced chromosomes of 1BL/1RS.

### QTL for yield and its response to N stress: their use in wheat molecular breeding programs

Alleles of QTL that increase YD in LN are of value in wheat breeding programs designed to increases NUE. In this study, *QYd-1D-1.1*, *QYd-1D-1.2*, *QYd-2D-1.1*, *QYd-2D-1.2*, *QYd-3A*, *QYd-4A-1*, *QYd-4A-2*, *QYd-4B-2*, *QYd-6B*, and *QYd-7B* showed positive additive effects that increase YD in LN, indicating that alleles from KN9204 of these QTL are elite alleles (Table 6). *QYd-1A-2.2*, *QYd-1B.1*, *QYd-4A.2*, *QYd-6A* and *QYd-7A.2* showed negative additive effects in LN, indicating that alleles from J411 of these QTL increase YD in LN (Table 6). Pyramiding these elite alleles in wheat molecular breeding programs might be an optimal war to improve NUE and thus increase YD potential under LN.

Low input agricultural practices, particularly LN input management requires varieties that are insensitive to N stress. YDDV could be used as a ‘global’ interaction variable to estimate the response of YD to N stress [21]. Decreasing alleles of QTL for YDDV can increase N-deficiency tolerance and thus can maintain yield under N stress. In this study, there were two and ten QTL with favorable alleles that decrease YDDV from KN9204 and J411, respectively. Therefore, we should pyramid these elite alleles in wheat molecular breeding programs to improve N stress tolerance.

**Figure 2:**
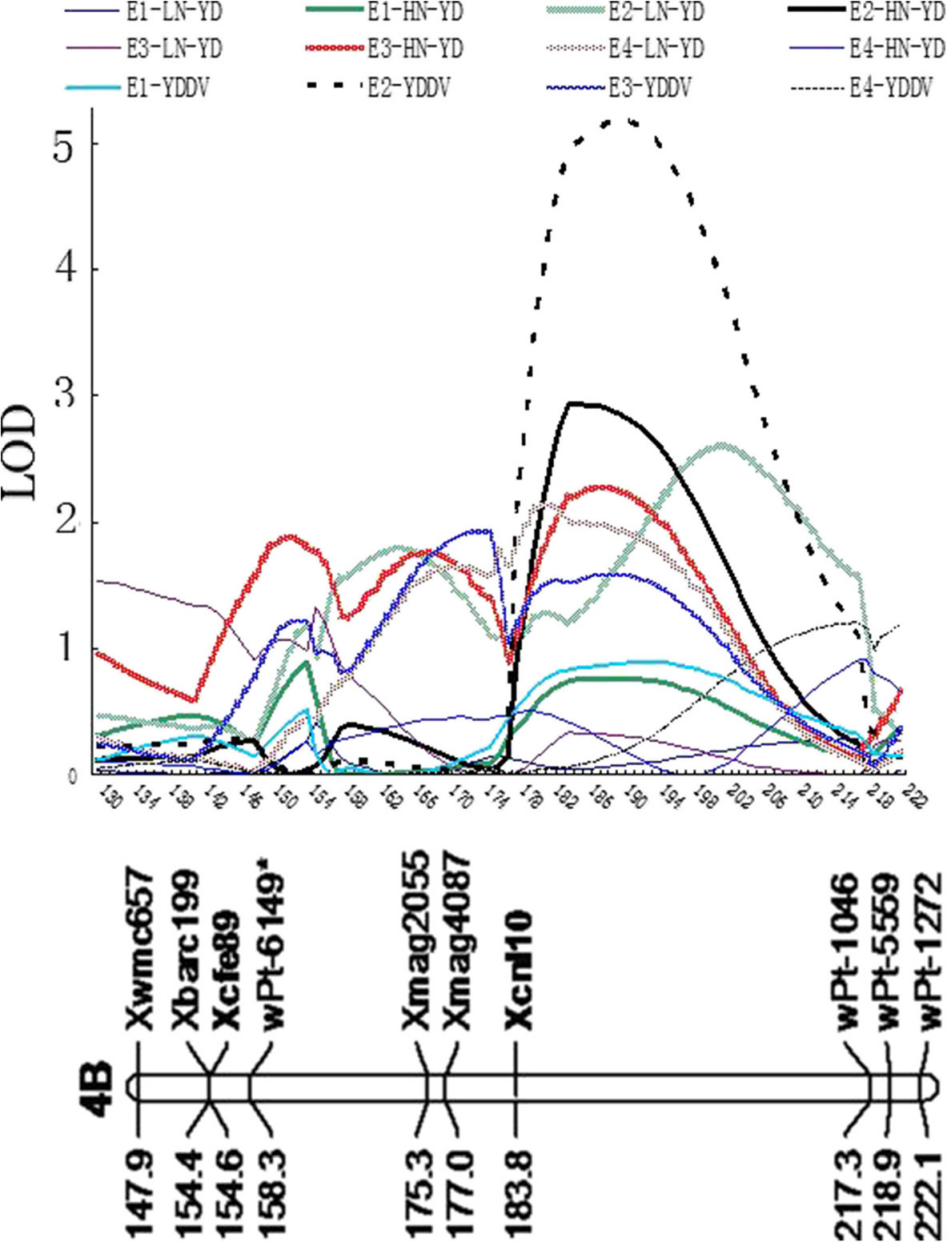
**The LOD value scanning of major stable QTL *QYd-4B-2 *** **and *QYddv-4B *** **in the eight individual environments.**

The conserved genomic regions harboring major stable QTL are valuable for MAS in breeding programs. *QYd-4B-2* and *QYddv-4B* shared support interval, and they both exhibited significance in multiple environments (Table 6, Figure 2). For YD and YDDV in all environments, the LOD curves peaked at approximately similar position of the *Xcnl10–wPt-1046* region, albeit some LOD values were relatively lower than the threshold (Figure 2). Interestingly, alleles of *QYd-4B-2* from KN9204 could increase YD in LN with positive additive effects of 0.62 g and 0.44 g; *QYddv-4B* from KN9204 with negative effect of −1.45 g and −0.45 g accounted for 19.09 and 6.87% of the phenotypic variance in E2 and E3, respectively. Unfortunately, limited polymorphic markers were available in this region; consequently, these two major stable QTLs resided on a relatively large confidence interval. More markers should be enriched in this region with the aim to finely map these QTLs and provide available markers for MAS. To date, information on the sequences of numerous bin-mapped wheat ESTs and scaffolds is publicly available [1,2] (http://wheat.pw.usda.gov/wEST/binmaps/). The relative physical bins of the *Xcnl10–wPt-1046* region are estimated in Figure 1, which will facilitate the fine mapping or even map-based cloning of this QTL.

## Conclusion

We developed a novel genetic map with g-SSR, e-SSR, DArT, STS, SRAP, ISSR, and morphological markers distributed across 21 wheat chromosomes spanning 3930.7 cM. The linear relationships between loci found in the present map and in previously compiled physical maps were specified. We also presented information on the genetic and physical positions and allele sizes (when possible) for 17 DArT, 50 e-SSR, 44 SRAP, five ISSR, and two morphological markers. Seven SDRs were identified on chromosomes 1B, 3BL, 4AL, 6AS, 6AL, 6BL, and 7B. A total of 22 and 12 QTL for YD and YDDV were identified, respectively. Of these, *QYd-4B-2* and *QYddv-4B*, two major stable QTL, shared support interval with alleles from KN9204 increasing YD in LN and decreasing YDDV. We probe into the use of these QTL in wheat breeding programs. In addition, factors affecting the occurrence of SDRs and the total map length were discussed in depth. This novel map facilitates the use of novel markers in wheat molecular breeding programs and genomics research. Moreover, QTL for YD and YDDV provide useful markers for wheat molecular breeding programs designed to increase yield potential under N stress.

The data sets supporting the results of this article are included within the article and its Additional file 1.

## Abbreviations

QTL: Quantitative trait loci; YD: Yield per plant; YDDV: Yield difference between the value under high nitrogen treatment and the value under low nitrogen treatment; g-SSR: Genomic simple sequence repeat; e-SSR: Expressed sequence tag-derived microsatellite; DArT: Diversity arrays technology; STS: Sequence-tagged sites; SRAP: Sequence-related amplified polymorphism; ISSR: Inter-simple sequence repeat; N: Nitrogen; HN: High nitrogen treatment; LN: Low nitrogen treatment; RIL: recombinant inbred line population; KN9204: Kenong9204; J411: Jing411; KJ-RILs: Recombinant inbred line population derived from the cross between Kenong9204 and Jing411; ORFs: Open reading frames; MAS: Marker-assisted selection; SDR: Segregation distortion region; NUE: Nitrogen use efficiency; LOD: Log-of-odds.

## Competing interests

The authors declare that they have no competing interests.

## Author’ contributions

CF, FXL, ZCH, and CM performed the genotyping and phenotyping of the parental lines and KJ-RILs. CF and FXL performed the linkage analysis and constructed the map. CF drafted the manuscript, and LJM, JJ, and ZW provided a critical review of the manuscript. LJM and JJ constructed the KJ-RIL population. All authors approved the final version of the manuscript.

## Supplementary Material

Additional file 1: Table S1 Polymorphic primer sequences for ISSR and SRAP markers. **Table S2.** Summary of the year, location and nitrogen treatment conditions in our study. **Table S3.** Density and distribution of markers in the novel genetic map of wheat. **Table S4.** Marker loci with distorted segregation and their distribution in the wheat genome. **Table S5.** Distribution of seven distorted chromosomal regions. **Table S6.** A comparison of map lengths (cM) of various linkage maps of the wheat genome generated in different mapping populations. Click here for file
