# Supplementary material for: A novel genetic map of wheat: utility for mapping QTL for yield under different nitrogen treatments
Source: BMC Genet. 2014 May 15;15:57. doi: 10.1186/1471-2156-15-57 (PMC4038385; doi:10.1186/1471-2156-15-57)
Supplement: Additional file 1: Table S1 — Polymorphic primer sequences for ISSR and SRAP markers. Table S2. Summary of the year, location and nitrogen treatment conditions in our study. Table S3. Density and distribution of markers in the novel genetic map of wheat. Table S4. Marker loci with distorted segregation and their distribution in the wheat genome. Table S5. Distribution of seven distorted chromosomal regions. Table S6. A comparison of map lengths (cM) of various linkage maps of the wheat genome generated in different mapping populations. [file 1471-2156-15-57-S1.doc]

**A novel genetic map of wheat:** **utility** **for** **mapping QTL for yield under different nitrogen treatments**

Fa Cui, Xiaoli Fan, Chunhua Zhao, Wei Zhang, Mei Chen, Jun Ji and Junming Li

**Additional file 1**

**Table S1** **Polymorphic primer sequences for ISSR and SRAP markers……………………………………………..2**

**Table S2 Summary of the year, location and nitrogen treatment conditions in our study…………………………3**

**Table S3** **Density and distribution of markers in the novel genetic map of wheat………………………….………4**

**Table S4 Marker loci with distorted segregation and their distribution in the wheat genome…………….………5**

**Table S5** **Distribution of seven distorted chromosomal regions………………………….…………………….…….6**

**Table S6 A comparison of map lengths (cM) of various linkage maps of the wheat genome generated in different mapping populations…………………………………………………………………………………………………….7**

**Table S1** **Polymorphic primer sequences for ISSR and SRAP markers**

| ***Markers*** | ***Primer sequences (5' →3')*** | ***Marker*** | ***Primer sequences (5' →3')*** |
| --- | --- | --- | --- |
| Me4 | TGAGTCCAAACCGGACA | Em7 | GACTGCGTACGAATTATG |
| Me6 | TGAGTCCAAACCGGGCT | Em10 | GACTGCGTACGAATTAGC |
| Me5 | TGAGTCCAAACCGGGAT | Em11 | GACTGCGTACGAATTACG |
| Me7 | TGAGTCCAAACCGGTAA | Em12 | GACTGCGTACGAATTTAG |
| Me9 | TGAGTCCAAACCGGAAT | Em13 | GACTGCGTACGAATTTCG |
| Me10 | TGAGTCCAAACCGGACC | Em15 | GACTGCGTACGAATTGGT |
| Me11 | TGAGTCCAAACCGGAAGC | Em19 | GACTGCGTACGAATTCAA |
| Me12 | TGAGTCCAAACCGGTAG | Em20 | GACTGCGTACGAATTCGA |
| Me13 | TGAGTCCAAACCGGTTG | Em21 | GACTGCGTACGAATTGAT |
| Me16 | TGAGTCCAAACCGGTAC | Em22 | GACTGCGTACGAATTCCT |
| Me18 | TGAGTCCAAACCGGAAG | Em23 | GACTGCGTACGAATTGAG |
| Me20 | TGAGTCCTTTCCGGTCC | Em25 | GACTGCGTACGAATTTCA |
| Me23 | TGAGTCCAAACCGGACG | Em26 | GACTGCGTACGAATTAAT |
| Me26 | TGAGTCCAAACCGGAAA | ISSR807 | AGAGAGAGAGAGAGAGT |
| Em2 | GACTGCGTACGAATTTGC | ISSR811 | GAGAGAGAGAGAGAGAC |
| Em3 | GACTGCGTACGAATTGAC | ISSR849 | GTGTGTGTGTGTGTGTYA |

Relevant information on SRAPs was obtained from an article written by Li and Quiros [1], and ISSR from that by Nagaoka T and Ogihara [2]

**Table S2** Summary of the year, location and nitrogen treatment conditions in our study

| En.^a^ | Year | Location | Soli nitrate-N contents (mg kg^-1^) | Soli total-N contents (mg kg^-1^) |
| --- | --- | --- | --- | --- |
| E1-LN | 2011-2012 | Shijiazhuang | 10.6 | 82.2 |
| E1-HN | 2011-2012 | Shijiazhuang | 43.1 | 121.3 |
| E2-LN | 2012-2013 | Shijiazhuang | 21.8 | 81.3 |
| E2-HN | 2012-2013 | Shijiazhuang | 44.6 | 128.6 |
| E3-LN | 2012-2013 | Beijing | 20.4 | 91.3 |
| E3-HN | 2012-2013 | Beijing | 38.4 | 122.5 |
| E4-LN | 2012-2013 | Xinxiang | 29.3 | 103.6 |
| E4-HN | 2012-2013 | Xinxiang | 38.6 | 122.9 |

^a^ En.=environments

**Table S3** **Density and distribution of markers in the novel genetic map of wheat**

| ***Chromosome*** | ***Total Markers*** | ***DarT*** | ***PCR*** | ***Map length***  ***(cM)*** ***^a^*** | ***Marker density***  ***(cM/Marker)*** |
| --- | --- | --- | --- | --- | --- |
| 1A | 29 | 15 | 14 | 246.7 | 8.5 |
| 1B | 61 | 35 | 26 | 189.1 | 3.1 |
| 1D | 14 | 5 | 9 | 102.6 | 7.3 |
| 2A | 29 | 5 | 24 | 247.9 | 8.5 |
| 2B | 36 | 18 | 18 | 188.1 | 5.2 |
| 2D | 47 | 14 | 33 | 351.8 | 7.5 |
| 3A | 24 | 12 | 12 | 121.2 | 5.1 |
| 3B | 54 | 29 | 24 | 242.4 | 4.5 |
| 3D | 20 | 15 | 5 | 145.9 | 7.3 |
| 4A | 35 | 16 | 19 | 252.9 | 7.2 |
| 4B | 19 | 6 | 12 | 176.0 | 9.3 |
| 4D | 2 | 0 | 2 | 35.2 | 17.6 |
| 5A | 8 | 2 | 6 | 46.3 | 5.8 |
| 5B | 18 | 12 | 6 | 107.1 | 6.0 |
| 5D | 6 | 0 | 6 | 46.8 | 7.8 |
| 6A | 38 | 24 | 14 | 127.8 | 3.4 |
| 6B | 49 | 32 | 17 | 323.8 | 6.6 |
| 6D | 11 | 2 | 9 | 143 | 13.0 |
| 7A | 35 | 13 | 22 | 295.8 | 8.5 |
| 7B | 28 | 17 | 11 | 244.3 | 8.7 |
| 7D | 28 | 15 | 13 | 296.0 | 10.6 |
| **Genome A** | 198 | 87 | 111 | 1338.6 | 6.8 |
| **Genome B** | 265 | 149 | 114 | 1470.8 | 5.6 |
| **Genome D** | 128 | 51 | 77 | 1121.3 | 8.8 |
| **Total** | 591 | 287 | 302 | 3930.7 | 6.7 |

^a^ The 31 gaps greater than 40 cM were excluded from the count of the total map length

**Table S4** Marker loci with distorted segregation and their distribution in the wheat genome

| ***Chr.*** | ***No. of skewed markers (P<0.05)*** | | | ***No. of skewed markers (P<0.01)*** | | |
| --- | --- | --- | --- | --- | --- | --- |
|  | **PCR** | **DArT** | **Total** | **PCR** | **DArT** | **Total** |
| 1A | 3 | 6 | 9 | 2 | 6 | 8 |
| 1B | 14 | 29 | 43 | 13 | 25 | 38 |
| 1D | 0 | 1 | 1 | 0 | 0 | 0 |
| 2A | 2 | 0 | 2 | 1 | 0 | 1 |
| 2B | 2 | 4 | 6 | 0 | 0 | 0 |
| 2D | 4 | 3 | 7 | 3 | 2 | 5 |
| 3B | 5 | 3 | 8 | 2 | 0 | 2 |
| 4A | 3 | 11 | 14 | 2 | 7 | 9 |
| 4B | 0 | 1 | 1 | 0 | 0 | 0 |
| 5A | 2 | 0 | 2 | 1 | 0 | 1 |
| 5B | 0 | 1 | 1 | 0 | 1 | 0 |
| 5D | 3 | 0 | 3 | 1 | 0 | 1 |
| 6A | 11 | 5 | 16 | 4 | 5 | 9 |
| 6B | 3 | 7 | 10 | 3 | 5 | 8 |
| 7A | 2 | 1 | 3 | 1 | 0 | 1 |
| 7B | 8 | 7 | 15 | 1 | 6 | 7 |
| Genome A | 23 | 23 | 46 | 11 | 18 | 29 |
| Genome B | 32 | 52 | 84 | 19 | 37 | 55 |
| Genome D | 7 | 4 | 11 | 4 | 2 | 6 |
| **Total** | 62 | 79 | 141 | 34 | 57 | 90 |
| %^a^ | 20.53 | 27.53 | 23.86 | 11.26 | 19.86 | 15.23 |

^a^ The percentage of skewed markers relative to the corresponding total number of markers listed in the above cells, i.e., PCR, DArT, and the total at the P<0.05 and P<0.01 levels.

**Table S5** Distribution of seven distorted chromosomal regions

| ***SDR*** | ***Interval*** | ***Chr.*** | ***No.*** |
| --- | --- | --- | --- |
| *SDR1* | *wPt-2751*–*Xwmc402.2* | 1B | 42 |
| *SDR2* | *wPt-8096*–*Xissr849* | 3BL | 4 |
| *SDR3* | *wPt-7064*–*Xgwm160* | 4AL | 14 |
| *SDR4* | *wPt-666574*–*wPt-730168* | 6AS | 4 |
| *SDR5* | *wPt-730772*–*wPt-729806* | 6AL | 6 |
| *SDR6* | *wPt-9930*–*Xcfe2* | 6BL | 7 |
| *SDR7* | *Xcfe100*–*wPt-9467* | 7B | 10 |

**Table S6 A comparison of map lengths (cM) of various linkage maps of the wheat genome generated in different mapping populations**

| **Population^a^** | **No. of loci** | **Type of marker** | **Total length (cM)** | **Reference** |
| --- | --- | --- | --- | --- |
| RIL | 279 | g-SSR | 3282 | [3] |
| RIL (T) | 259 | RFLP, etc | 1352 | [4] |
| RIL (T) | 306 | RFLP, g-SSR, AFLP, etc | 3598 | [5] |
| RIL | 436 | g-SSR, RFLP, AFLP | 2260 | [6] |
| DH | 659 | g-SSR | 3685 | [7] |
| RIL | 380 | RFLP , g-SSR, etc | 3086 | [8] |
| RIL | 584 | RFLP, AFLP, g-SSR, e-SSR | 4641 | [9] |
| DH, RIL | 1235 | g-SSR | 2569 | [10] |
| RIL | 591 | g-SSR | 4181 | [11] |
| DH | 900 | RAPD, g-SSR, AFLP | 3948 | [12] |
| RIL | 352 | g-SSR, TRAPs | 3045 | [13] |
| DH | 567 | RFLP, AFLP, g-SSR, etc | 3522 | [14] |
| RIL | 1406 | RFLP, g-SSR | 2654 | [15] |
| DH | 369 | g-SSR, e-SSR, etc. | 2793 | [16] |
| DH | 464 | g-SSR | 3441 | [17] |
| DH | 749 | g-SSR, DArT, RFLP, AFLP, etc | 2937 | [18] |
| DH | 624 | AFLP, g-SSR, DArT | 2596 | [19] |
| RIL | 381 | g-SSR, e-SSR, ISSR, SRAP, STS, etc | 3637 | [20] |
| RIL (D) | 179 | g-SSR, RFLP | 1262 | [21] |
| RIL | 1467 | g-SSR, RFLP, etc. | 5332 | [22] |
| DH, | 1644 | g-SSR, DArT, AFLP, RFLP | 2622 | [23] |
| DH | 305 | g-SSR, e-SSR | 2142 | [24] |
| RIL (T) | 554 | g-SSR, DArT | 2022 | [25] |
| RIL (T) | 690 | g-SSR, DArT | 2317 | [26] |
| RIL | 887 | RAPD, g-SSR, e-SSR, STS | 4223 | [27] |
| DH | 632 | g-SSR, TRAP, RFLP, etc | 3812 | [28] |
| RIL, DH | 385/575/275/468 | g-SSR, DArT | 3013/2825/2198/3058 | [29] |
| RIL | 214 | g-SSR, AFLP, etc. | 3972 | [30] |
| RIL (T) | 275 | g-SSR, e-SSR, etc. | 1605 | [31] |
| RIL | 719 | g-SSR, e-SSR, ISSR, SRAP, STS, DArT | 4008 | [32] |
| RIL (T) | 1479 | g-SSR, DArT, SNP | 2967 | [33] |
| RIL | 338/357 | g-SSR, e-SSR, ISSR, STS | 2856/3011 | [34] |
| RIL, F_2 :3_ (T) | 1898 | g-SSR, e-SSR, DArT, etc | 3058.6 | [35] |
| DH (T) | 588 | g-SSR, DArT, etc | 2048.8 | [36] |
| RIL | 1127 | g-SSR, DArT, e-SSR, ISSR, SRAP, STS | 2977 | [37] |
| RIL | 591 | g-SSR, DArT, e-SSR, ISSR, SRAP, STS | 3931 | Current map |

^a^ T: Tetraploid wheat mapping population with AABB genomes; D: Diploid wheat mapping population with an AA genome; the remaining maps represent allohexaploid bread wheat mapping population with AABBDD genomes.

**References**

1. Nagaoka T, Ogihara Y: **Applicability of inter-simple sequence repeat polymorphisms in wheat for use as DNA markers in comparison to RFLP and RAPD markers.** *Theor Appl Genet* 1997, **94:**597–602.
2. Li G, Quiros CF: **Sequence-related amplified polymorphism (SRAP), a new marker system based on a simple PCR reaction: its application to mapping and gene tagging in Brassica.** *Theor Appl Genet* 2001, 103:455–461.
3. Röder MS, Korzum V, Gill BS, Ganal MW. **The physical mapping of microsatellite markers in wheat.** *Genome* 1998, **41:** 278–283.
4. Blanco A, Bellomo MP, Cenci A, De Giovanni C, D'Ovidio R, Iacono E, Laddomada B, Pagnotta MA, Porceddu E, Sciancalepore A, Simeone R, Tanzarella OA: **A genetic linkage map of durum wheat.** *Theor Appl Genet* 1998, **97:**721–728.
5. Nachit MM, Elouafi I, Pagnotta MA, EI SA, Iacono E, Labhilili M, Asbati A, Azrak M, Hazzam H, Benscher D, Khairallah M, Ribaut J-M, Tanzarella OA, Porceddu E, Sorrells ME: **Molecular linkage map for an intraspecific recombinant inbred population of durum wheat** **(*Triticum turgidum* L. var. durum).** *Theor Appl Genet* 2001, **102:**177–186.
6. Groos C, Gay G, Perretant MR, Gervais L, Bernard M, Dedryver F, Charmet G: **Study of the relationship between pre-harvest sprouting and grain color by quantitative trait loci analysis in a white × red grain bread wheat cross.** *Theor Appl Genet* 2002, 104:39–47.
7. Sourdille P, Cadalen T, Guyomarc’h H, Snape JW, Perretant MR, Charmet G, Boeuf C, Bernard S, Bernard M: **An update of the Courtot×Chinese Spring intervarietal molecular marker linkage map for the QTL detection of agronomic traits in wheat.** *Theor Appl Genet* 2003, **106:**530–538.
8. Paillard S, Schnurbusch T, Winzeler M, Messmer M, Sourdille P, Abderhalden O, Keller B, Schachermayr: **An integrative genetic linkage map of winter wheat (*Triticum aestivum* L.).** *Theor Appl Genet* 2003, **107:**1235–1242.
9. Gao LF, Jing RL, Huo NX, Li Y, Li XP, Zhou RH, Chang XP, Tang JF, Ma ZY, Jia JZ: **One hundred and one new microsatellite loci derived from ESTs (E-SSR) in bread wheat.** *Theor Appl Genet* 2004, **108:**1392–1400.
10. Somers DJ, Isaac P, Edwards K: **A high-density microsatellite consensus map for bread wheat (*Triticum aestivum* L.).** *Theor Appl Genet* 2004, **109:**1105–1114.
11. Yu JK, Dake TM, Singh S, Benscher D, Li W, Gill B, Sorrells ME: **Development and mapping of EST-derived simple sequence repeat markers for hexaploid wheat.** *Genome* 2004, **47:**805–818.
12. Suenaga K, Khairallah M, William HM, Hoisington DA: **A new intervarietal linkage map and its application for quantitative trait locus analysis of ‘‘gigas’’ features in bread wheat.** *Genome* 2005, **48:**65–75.
13. Liu ZH, Anderson JA, Hu J, Friesen TL, Rasmussen JB, Faris JD: **A wheat intervarietal genetic linkage map based on microsatellite and target region amplified polymorphism markers and its utility for detecting quantitative trait loci.** *Theor Appl Genet* 2005, **111:**782–794.
14. Quarrie SA, Steed A, Calestani C, Semikhodskii A, Lebreton C, Chinoy C, Steele N, Pljevljakusić D, Waterman E, Weyen J, Schondelmaier J, Habash DZ, Farmer P, Saker L, Clarkson DT, Abugalieva A, Yessimbekova M, Turuspekov Y, Abugalieva S, Tuberosa R, Sanguineti MC, Hollington PA, Aragués R, Royo A, Dodig D: **A high-density genetic map of hexaploid wheat (*Triticum aestivum* L.) from the cross Chinese Spring × SQ1 and its use to compare QTLs for grain yield across a range of environments.** *Theor Appl Genet* 2005, **110:**865–880.
15. Song QJ, Shi JR, Singh S, Fickus EW, Costa JM, Lewis J, Gill BS, Ward R, Cregan PB: **Development and mapping of microsatellite (SSR) markers in wheat.** *Theor Appl Genet* 2005, **110:** 550–560.
16. McCartney CA, Somers DJ, Humphreys DG, Lukow O, Ames N, Noll J, Cloutier S, McCallum BD: **Mapping quantitative trait loci controlling agronomic traits in the spring wheat cross RL4452 × ‘AC Domain’.** *Genome* 2005, **48:**870–883.
17. Torada A, Koike M, Mochida K, Ogihara Y: **SSR-based linkage map with new markers using an intraspecific population of common wheat.** *Theor Appl Genet* 2006, **112:**1042−1051.
18. Akbari M, Wenzl P, Caig V, Carling J, Xia L, Yang S, Uszynski G, Mohler V, Lehmensiek A, Kuchel H, Hayden MJ, Howes N, Sharp P, Vaughan P, Rathmell B, Huttner E, Kilian: **A Diversity arrays technology (DArT) for high-throughput profiling of the hexaploid wheat genome.** *Theor Appl Genet* 2006, **113:**1409–1420.
19. Semagn K, Bjørnstad A, Skinnes H, Marøy AG, Tarkegne Y, William M: **Distribution of DArT, AFLP and SSR markers in a genetic linkage map of a double haploid hexaploid wheat population.** *Genome* 2006, **49:**545–555.
20. Li SS, Jia JZ, Wei XY, Zhang XC, Li LZ, Chen HM, Fan YD, Sun HY, Zhao XH, Lei TD, Xu YF, Jiang FS, Wang HG, Li LH：**A intervarietal genetic map and QTL analysis for yield traits in wheat.** *Mol Breeding* 2007，**20:**167–178.
21. Singh K, Ghai M, Garg M, Chhuneja P, Kaur P, Schnurbusch T, Keller B, Dhaliwal HS: **An integrated molecular linkage map of diploid wheat based on a *Triticum boeoticum* × *T. monococcum* RIL population.** *Theor Appl Genet* 2007, **115:**301–312.
22. Ganal MW, Röder MS: **Microsatllite and SNP markers in wheat breeding.** *Genomics Applications in Crops* 2007, **2:**1–24.
23. Crossa J, Burgueño, Dreisigacker S, Vargas M, Herrera-Foessel SA, Lillemo M, Singh RP, Trethowan R, Warburton M, Franco J, Reynolds M, Crouch JH, Ortiz R: **Association analysis of historical bread wheat germplasm using additive genetic covariance of relatives and population structure**. *Genetics* 2007, **177:** 1889–1913.
24. Zhang KP, Zhao L, Tian JC, Chen GF, Jiang XL, Liu B: **A genetic map constructed using a doubled haploid population derived from two elite Chinese common Wheat varieties.** *J integr plant biol* 2008, **50:**941–950.
25. Mantovani P, Maccaferri M, Sanguineti MC, Tuberosa R, Catizone I, Wenzl P, Thomson B, Carling J, Huttner E, Ambrogio ED, Kilian A : **An integrated DArT-SSR linkage map of durum wheat.** *Mol Breed* 2008, **22:**629–648.
26. Peleg Z, Saranga Y, Suprunova T, Ronin YW, Röder MS, Kilian A, Korol AB, Fahima T: **High-density genetic map of durum wheat × wild emmer wheat based on SSR and DArT markers.** *Theor Appl Genet* 2008, **117:**103–115.
27. Xue S, Zhang ZZ, Lin F, Kong ZX, Cao Y, Li CJ, Yi HY, Mei MF, Zhu HL, Wu JZ, Xu HB, Zhao DM, Tian DG, Zhang CQ, Ma ZQ: **A high-density intervarietal map of the wheat genome enriched with markers derived from expressed sequence tags.** *Theor Appl Genet* 2008, **117:**181–189.
28. Chu CG, Xu SS, Friesen TL, Faris JD: **Whole genome mapping in a wheat doubled haploid population using SSRs and TRAPs and the identification of QTL for agronomic traits.** *Mol Breeding* 2008, **22:**251–266.
29. Francki MG, Walker E, Crawford AC, Broughton S, Ohm HW, Barclay I, Wilson RE, McLean R: **Comparison of genetic and cytogenetic maps of hexaploid wheat (*Triticum aestivum* L.) using SSR and DArT markers.** *Mol Genet Genomics* 2009, **281:**181–191.
30. Mohan A, Kulwal P, Singh R, Kumar V, Mir RR, Kumar J, Prasad M, Balyan HS, Gupta PK: **Genome-wide QTL analysis for pre-harvest sprouting tolerance in bread wheat.** *Euphytica* 2009, **168:**319–329.
31. Gadaleta A, Giancaspro A, Giove SL, Zacheo S, Mangini G, Simeone R, Signorile A, Blanco A: **Genetic and physical mapping of new EST-derived SSRs on the A and B genome chromosomes of wheat.** *Theor Appl Genet* 2009, **118:**1015–1025.
32. Wang YY, Sun XY, Zhao Y, Kong FM, Guo Y, Zhang GZ, Pu YY, Wu K, Li SS: **Enrichment of a common wheat genetic map and QTL mapping for fatty acid content in grain.** *Plant Sci* 2011, **181:**65–75.
33. Trebbi D, Maccaferri M, de Heer P, Sørensen A, Giuliani S, Salvi S, Sanguineti MC, Massi A, Vossen EAG, Tuberosa R: **High-throughput SNP discovery and genotyping in durum wheat (*Triticum durum* Desf.).** *Theor Appl Genet* 2011, **123:**555–569.
34. Cui F, Ding AM, Li J, Zhao CH, Wang L, Wang XQ, Qi XL, Li XF, Li GY, Gao JR, Wang HG: **QTL detection of seven spike-related traits and their genetic correlations in wheat using two related RIL populations.** *Euphytica* 2012, **186:**177–192.
35. Marone D, Laidò G, Gadaleta A, Colasuonno P, Ficco DBM, Giancaspro A, Giove S, Panio G, Russo MA, Vita PD, Cattivelli L, Papa R, Blanco A, Mastrangelo AM: **A high-density consensus map of A and B wheat genomes.** *Theor Appl Genet* 2012, **106:**1619–1638.
36. Zhang L, Luo JT, Hao M, Zhang LQ, Yuan ZW, Yan ZH, Liu YX, Zhang B, Liu BL, Liu CJ, Zhang HG, Zheng YL, Liu DC: **Genetic map of *Triticum* turgidum based on a hexaploid wheat population without genetic recombination for D genome.** *BMC Genet* 2012, **13:**69. doi: 10.1186/1471-2156-13-69
37. Cui F, Zhao CH, Ding AM, Li J, Wang L, Li XF, Bao YG, Li JM, Wang HG: **Construction of an integrative linkage map and QTL mapping of grain yield-related traits using three related wheat RIL populations.** *Theor Appl Genet* 2014, **127:**659–675.
